# Private renters in shared housing: investigating housing conditions and mental well-being in Australia during COVID-19

**DOI:** 10.1007/s10901-023-10038-w

**Published:** 2023-05-20

**Authors:** Piret Veeroja, Zoë Goodall, Nestor Agustin Guity-Zapata, Wendy Stone

**Affiliations:** Centre for Urban Transitions, School of Social Sciences, Media, Film and Education, Swinburne University of Technology, Melbourne, Australia

**Keywords:** Rental housing, Isolation, Anxiety, Worry, Shared housing, COVID-19

## Abstract

Lockdowns were the major policy response to COVID-19 containment in many countries, and subsequently many people spent abnormal amounts of time at home. Research has found that housing conditions affected more peoples’ mental health during the COVID-19 crisis than prior to it, and vulnerable groups were especially affected. One group that may be particularly vulnerable is private renters in shared housing. Using a socio-economic lens, our research examined to what extent mental well-being outcomes were associated with housing conditions in shared housing under COVID-19 restrictions in Australia. Data about private renters were obtained from the Australian Rental Housing Conditions Dataset (*n* = 1908), collected in mid-2020 during the easing of the first lockdown restrictions. Respondents living in shared arrangements reported higher levels of worry and anxiety (8.5–13.2%) and loneliness and isolation (3.7–18.3%) compared to other household types. Binary logistic regressions showed that COVID-19-related mental and financial well-being variables were the main contributors in COVID-19-related worry/anxiety and loneliness/isolation models. Accumulated housing problems were the only housing condition measure that was significant in the worry/anxiety model. Participants who had more than two people living in a household felt 1.4 times lonelier/isolated compared to those who lived with four or more people. Males and participants who reported good mental health were less likely to feel COVID-19-related worry/anxiety and loneliness/isolation. Our analysis demonstrates the importance of measures for mental health and income during a pandemic and concludes with recommendations of support for shared housing renters during and beyond crisis events.

## Introduction

Lockdowns and ‘stay at home’ orders were the main policy responses to the COVID-19 pandemic in many countries, including Australia. These policies, however, did not consider housing inequities, varying housing conditions, energy poverty, overall social hardship and vulnerability (Horne et al., 2021). Indeed, recent studies have concluded that housing conditions were significant to people’s mental well-being during the pandemic (Bower et al., 2021; Horne et al., 2021; Preece et al., 2021). Previous studies have established that vulnerable groups, such as young adults (Churchill, 2021; Nunes et al., 2021), females (Kugler et al., 2021), people with low educational qualifications (Kikuchi et al., 2021; Kugler et al., 2021; Nunes et al., 2021), people with low incomes, unstable jobs and few savings (Baker et al., 2022; Kikuchi et al., 2021), people who are culturally and linguistically diverse (Mude et al., 2021), and people with smaller social networks (Bryant et al., 2016), faced amplified social and economic effects of the pandemic (Raynor & Panza, 2021). People living in shared housing—usually defined as living with people who are not (all) family members or partners (Heath et al., 2017; McNamara & Connell, 2007)—are often associated with some of these vulnerabilities (Raynor & Panza, 2021). Additionally, financial and housing pressures due to the COVID-19 crisis may have driven more people into shared living arrangements (Vandenberg, 2021). Shared housing has different forms, including friends living together, strangers cohabiting after connecting through online platforms, and housing cooperatives, and it is no longer a marginal living arrangement (Clark et al., 2017; Druta et al., 2021; Maalsen, 2019; Parkinson et al., 2021). There is growing literature that investigates how this pandemic has affected mental health for various groups, and a small body of research examines the specific pandemic experiences of people in shared housing (see Blanc & Scanlon Bradley, 2022; Buckle et al., 2022; Raynor & Frichot, 2022; Raynor & Panza, 2020, 2021). Adding to this body of work, our paper focuses on the relationship between housing conditions and mental well-being for people living in shared housing.

Living in shared housing may increase or decrease mental well-being outcomes. Chen and Wang (2021) found that participants living in two to four people households had better mental well-being compared to people living alone during COVID-19 lockdowns in the UK, however, crowded households with five or more people had a negative impact on mental well-being. Indeed, higher household density (less space per person) has been found to decrease people’s mental health during the pandemic (Groot et al., 2020). Following Dupuis and Thorns (1998), some feminist care scholars have stressed that security, familiarity, control, intimacy, comfort and personal expression make a dwelling a home regardless of cultural and social backgrounds (Bowlby & Jupp, 2021; Boccagni & Kusenbach, 2020: 8), however achievement of these features depends on other residents (Bowlby & Jupp, 2021). This means that sharing a dwelling may contribute to the extent a person feels at home, especially when more people than usual are using the space. Moreover, many people were asked to work from home or support children attending school via home-based online learning, but homes (and especially shared homes) might not be suitably equipped due to insufficient space and quiet areas (Bowlby & Jupp, 2021). COVID-19-related changes might blur the boundaries between home, work and leisure-related spaces for shared housing residents (Druta et al., 2021). Conversely, based on studies by Chen and Wang (2021), Szkody et al. (2021) and Raynor and Frichot (2022), and given that lockdowns often prohibited people from seeing friends and family, shared housing may have alleviated loneliness if tenants experienced social interaction, care and support with their co-residents.

This paper thus aims to investigate a) whether private renters in shared living arrangements had better or worse mental well-being outcomes during the COVID-19 pandemic compared with other household types, and b) to what extent housing conditions contributed to mental well-being outcomes in shared living arrangements. Poor rental housing conditions undermine tenants’ feelings of home, which in turn, have negative effects on mental well-being (Garnham et al., 2022). Given the increased time at home and mental health impacts of the pandemic, it is important to study how the impact of housing conditions may be exacerbated by this major event. Our interest is in housing *conditions*; however, it is well established that housing affordability, security, crowding and meanings around home are bundled together when considering well-being benefits (Bratt, 2002), and the effect of housing conditions and these attributes to mental wellbeing were found to be amplified during the pandemic (Waldron, 2022). Therefore, we also consider these housing attributes in our study. This study will contribute to the literature by understanding how housing supports mental well-being in crisis situations and help to prepare for future crisis responses with special focus on vulnerable groups. While other studies have explored housing conditions and wellbeing during the pandemic for a range of household and tenure types (Akbari et al., 2021; Bower et al., 2021; Horne et al., 2021), we look specifically at private renters in shared living situations. Similarly to other studies of shared housing during COVID-19, we examine how a shared living environment may specifically impact people’s experiences of the pandemic, and the policy implications of the results.

Our study draws on the Australian Rental Housing Conditions Dataset (ARCHD) which included a dedicated COVID-19 focused module (Baker et al., 2020a). In home-ownership societies, rental tenures are considered second-best in housing aspirations literature (Stone et al., 2020) and are associated with tenure insecurity, affordability concerns (Hulse & Milligan, 2014; Kemp, 2002; Pawson et al., 2017) and lower health outcomes (Baker et al., 2020b; Schafer et al., 2022). Additionally, renters are less likely to be able to undertake home modifications to improve energy efficiency or comfort, including during stay-at-home rules (Horne et al., 2021). While our data is solely from Australia, and Australia’s coronavirus response was shaped by specific national factors (e.g. closing international borders for non-residents early), results are relevant in an international context given the increased importance of private rental in traditional home-ownership dominated societies (Hulse et al., 2019) and the increase in shared living in multiple countries across the globe (Bricocoli & Sabatinelli, 2016; Heath et al., 2017; Maalsen, 2019). Data used in this study was collected in July–August 2020, shortly after the first nationwide lockdown in Australia had ended. From mid-2020 onwards, Australia experienced more outbreaks and lockdowns, but these were generally confined to individual states, as state leaders implemented strong interstate borders.

## Background

### Shared living in Australia

Living in shared housing is increasing in Australia, as in many other countries. Housing unaffordability and changing life-course trajectories are credited with influencing the uptake of shared housing, particularly for young adults, in Australia (McNamara & Connell, 2007), as well as parts of Europe (Bricocoli & Sabatinelli, 2016; Heath et al., 2017; Kenyon & Heath, 2001) and Asia (Cho et al., 2019; Druta & Ronald, 2021), although the standard dwelling type and the normalisation of shared housing can vary greatly between countries. Researchers have noticed the shift from ‘Generation Rent’, reflecting young adults who live in the private rental sector without necessarily transitioning to ownership, to ‘Generation Share’, reflecting the increasing importance of shared housing in young age (Maalsen, 2019; Uyttebrouck et al., 2020). Shared housing is also an alternative to social housing that is criticised due to declining stock and long waiting lists (Baker et al., 2020b; Clarke et al., 2020; Horne et al., 2021; Hulse & Milligan, 2014; Kemp, 2002; Pawson et al., 2017; Schafer et al., 2022; Stone et al., 2020). In Australia, latest Census data show that  3.9% of households were ‘group households’ (Australian Bureau of Statistics, 2022). The Australian Census’s definition of ‘group household’ excludes houses containing couple or family relationships (Australian Bureau of Statistics, 2021) meaning the actual number of shared houses could be underestimated (McNamara & Connell, 2007). Liu et al. (2020) and others have, in contrast, illuminated multi-generational housing as a form of house-share in Australia and similar countries. Estimates of multi-generational living in Australia suggest multiculturalism and housing affordability pressures are driving an increase of this household form, however multigenerational families (included in the national statistics as ‘other family’) remain a small minority, accounting for only 1.8 per cent of family households in 2016 (Australian Bureau of Statistics, 2019).

The Australian Rental Housing Conditions Dataset (ARHCD) (Baker et al., 2022) utilised in this study, used the term ‘shared living arrangement’ when asking participants about their household structure. We theorise that people who selected ‘shared living arrangement’ could include those in share-houses, in housing cooperatives, cohousing or in multi-family or intergenerational households—in short, those who did not fit into the categories of couple with no children, couple with children, one parent family with children, single person living alone, or other. This is the reason ‘shared living’ or ‘shared living arrangement’ is used in describing empirical parts of this research.

### Shared housing and mental well-being

Research has shown that there can be many benefits and positive emotions associated with living in shared housing. These include saving money, social connections, and reduced time on housework (Clark et al., 2017; Heath, 2004; Kim & Yoon, 2010; Maalsen, 2019; McNamara & Connell, 2007). However, there can also be negative impacts from living in shared housing. While conflict is an inevitable part of shared living (Clark et al., 2020; Liang, 2018), more severe feelings of emotional distress can occur. A lack of choice in sharing housing could cause feelings of loneliness, isolation and anxiety, especially when sharers do not know each other (Nasreen & Ruming, 2021). In Wilkinson and Ortega-Alcázar (2019) and Ortega-Alcázar and Wilkinson (2021) research with young welfare recipients in shared housing in UK, women, people of colour and LGBTQ+ tenants reported feeling trapped, anxious and in fear of their housemates.

Within the stressful conditions of living through a pandemic and economic downturn where people are instructed to ‘stay at home’, these negative impacts of shared housing could be exacerbated. A growing body of work has examined pandemic impacts specific to people living in shared housing and found generally negative outcomes. Specific to COVID-19, Raynor and Panza (2021: 6) in a study of shared housing tenants in Victoria, Australia found that 50% of their sample ‘experienced a deterioration in mental health’. Their sample also suffered work-related shocks including losing their job or having reduced hours, and for 47% of participants, their financial situation became ‘worse or dramatically worse’ (Raynor & Panza, 2021: 6). Raynor and Panza (2021: 2) point out that share-house tenants may have varying ability to access government support measures if their lease arrangements were informal. Additionally, they suggest that share-house tenants may experience tension because finances are generally not shared, unlike in family households, which could lead to disparate financial impacts (Raynor & Panza, 2021: 2). Buckle et al. (2022), using data from shared housing advertisements in Sydney, Australia, found overcrowded housing being advertised, which raised clear health risks including contracting COVID-19. Blanc and Scanlon Bradley (2022), surveying people in shared housing in London, UK, found people struggled to create adequate work-from-home environments, and experienced increased challenges of living with other people. Raynor and Frichot (2022), drawing from interviews in Melbourne, Australia during lockdown, found that people living in share-houses enacted networks of care with their housemates, but also experienced challenges within the household and when engaging with property managers and government support. It is this burgeoning body of work on shared housing and COVID-19 to which we contribute.

### Australian policy responses to COVID-19

**Fig. 1:**
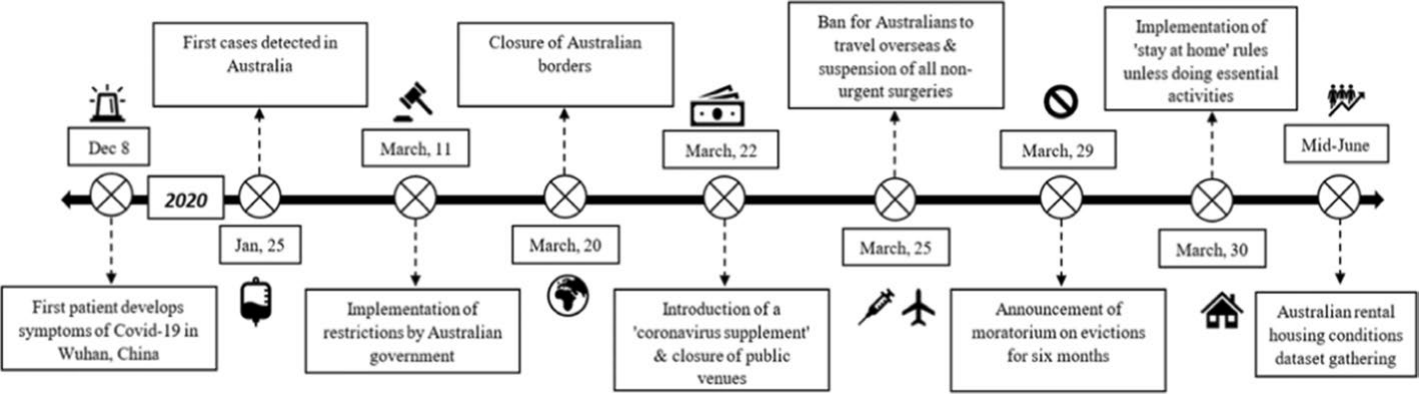
Timeline of the first lockdown in Australia (Source: Authors, drawing from Australian Government press releases collated at Campbell and Vines (2021))

Coronavirus cases began to appear in Australia in January 2020, and many federal government measures and restrictions were implemented in March 2020. These included the closure of public venues, banning people from entering or leaving the country, and instructing people to stay at home unless doing essential activities (Campbell & Vines, 2021), see Fig. 1. Support measures introduced included eviction moratoriums and a ‘coronavirus supplement’ welfare payment (Leishman et al., 2022). The gradual removal of some of these restrictions was announced by the Australian Government on 8 May 2020 (Campbell & Vines, 2021), and data used in this study was collected in July–August 2020, shortly after this. From mid-2020 onwards, Australia experienced more significant outbreaks and lockdowns, but these were confined to individual states, rather than national in scope.

International evidence shows that housing issues during the pandemic increased or at least were more evident, given the identification or appearance of stress, anxiety, and depression problems (Baker et al., 2020b; Ozamiz-Etxebarria et al., 2020). Indeed, Newton et al. (2022) found that enduring poor housing conditions affected renters’ mental wellbeing during the pandemic and led to anxiety and depression. Before the pandemic even began, the current and growing proportion of the Australian population renting, whether in the private or social sector, was flagged as a challenge requiring special attention (Burke et al., 2020; Productivity Commission, 2019). A recent reflection on the efficacy of the combination of housing measures in years one and two of the pandemic in Australia highlights both the range of measures implemented (income support, rental support measures, evictions moratoria, homeowner assistance) as well as the relative precarity of the private rental sector, for which many measures were implemented to protect tenants and investor landlords (Leishman et al., 2022). Recent Australian studies of renters during the pandemic highlight tenants’ stress, anxiety, economic insecurity and housing struggles, especially for specific groups like temporary visa-holders and those in informal housing (Buckle et al., 2020; Morris et al., 2021; Oswald et al., 2022). The challenges of private renting in Australia pre-pandemic, and the precarity and other housing problems that evidently increased during the pandemic, indicate that there is further need to study how renters were impacted.

## Methodology

### The Australian Rental Housing Conditions Dataset

The Australian Rental Housing Conditions Dataset (ARCHD) (Baker et al., 2020a) was used to capture the relationship between housing conditions, affordability, security and COVID-19-related well-being in shared living arrangements. Data were collected from July–August 2020 independently of this study. This time period was after the first round of COVID-19-related lockdowns and restrictions in Australia. The survey was carried out nationally and included measures of housing conditions, affordability, tenure security, health and well-being, but also a COVID-19 module that captured participants’ financial and mental well-being and housing suitability. Quotas of age (older than 18 years), tenure (social renters and private renters) and State/Territory were used. Overall, 13,597 participants responded to the survey via web-based self-administered questionnaires. Steps were taken during data collection to ensure spatial and tenure representation, with sampling from discrete households. Given the highly dynamic pandemic rental context in which data were collected it is theoretically possible (although highly unlikely) that two responses came from the same household and responses are treated as representing discrete household/dwelling units.

Participants who responded that their household structure was a shared living arrangement in private rental housing were considered in this study (*n* = 1908).

### Independent variables

#### Housing conditions

Our literature review found that housing conditions measures varied from study to study mainly because there is no agreement on which housing conditions should be included. Therefore, five groups of housing conditions variables were distinguished from the literature which were able to be investigated using the ARHCD: 1) housing problems (i.e. Pevalin et al., 2017); 2) thermal comfort (i.e. Liddell & Guiney, 2015); 3) noise (i.e. Dzhambov et al., 2018); 4) safety; and 5) overall perception of housing (i.e., Wells & Moch, 2003).

The survey captured whether participants had problems with dampness, mould, cracks in walls/floors, sinking or moving foundations, walls/windows/floors not levelled, wood rot or termite damage, electrical problems, roof defect and plumbing. The number of problems per participant were counted for the purposes of this study. Safety was captured by asking whether participants’ home had a functioning smoke detector, deadlocks on all external doors, locking mechanisms on windows, a security alarm and security screens on doors and windows. The number of security measures per participant were counted for the purposes of this study. Measures and measurement scales are shown in Table 3.

#### Housing attributes

Housing affordability was measured by asking participants whether they considered rent affordable. Unaffordable housing has been related with poorer mental well-being outcomes, and especially in rental housing (Mason et al., 2013; Waldron, 2022). Years in building was used as a proxy for tenure security. This measure was chosen over ‘length of current lease agreement’ as people living in shared houses often do not have a formal lease agreement (Buckle et al., 2022). A recent longitudinal study by Li et al. (2022) found that initially private renters had worse mental health outcomes compared to homeowners, however, renters who resided more than six years in the same address had similar mental health outcomes as homeowners. Other recent studies have found that tenure security had a negative impact on private renters’ mental well-being, see Waldron (2022) and Brown et al. (2022). Dwelling type and number of bedrooms were used as a proxy for size of home; number of adults and number of children in household as proxies for crowding in shared living arrangement. Measures and measurement scales are shown in Table 3.

#### COVID-19-related financial and mental well-being

The survey measured whether participants had withdrawn their superannuation (funds paid to all workers that are usually inaccessible until retirement), used savings, received government assistance and undertook additional work as a result of the COVID-19 crisis to reflect participants’ financial stress. In terms of housing, the survey asked whether participants’ rent had become unaffordable or they had to look for housemates as a result of the crisis.

To capture mental well-being, participants were asked if they felt higher levels of worry/anxiety, difficulties in personal relationships, experienced helplessness, had lowered self-esteem or change in mental health as a result of the crisis. Measures and measurement scales are shown in Table 3.

#### Covariates

Age group, gender and perceived mental health were entered as covariates to the modelling. Measures and measurement scales are shown in Table 2.

### Dependent variables

#### COVID-19-related well-being outcomes

COVID-19-related worry/anxiety and loneliness/isolation represented COVID-19-related mental well-being outcomes in this study. Loneliness is a distressing feeling when a quantity or quality of a person’s social needs are not met (Hawkley & Cacioppo, 2010) and depends on individuals personal social needs. We note that loneliness and isolation are often considered as different constructs in well-being studies. However, Hawkley and Cacioppo (2010) found that *perceived* social isolation and loneliness are synonyms. All measures in our study are respondents’ perceptions. We also note that worry and anxiety typically refer to different constructs in well-being studies. However, some COVID-19-related studies have considered these together as a proxy for COVID-19-related fear (Solymosi et al., 2021); an approach adopted in the present study.

ARHCD survey participants were asked whether they had experienced (a) higher level of worry and anxiety and (b) loneliness and isolation, as a result of COVID-19. Both were binary variables measured as a yes/no dichotomy.

### Analytic strategy

**Fig. 2:**
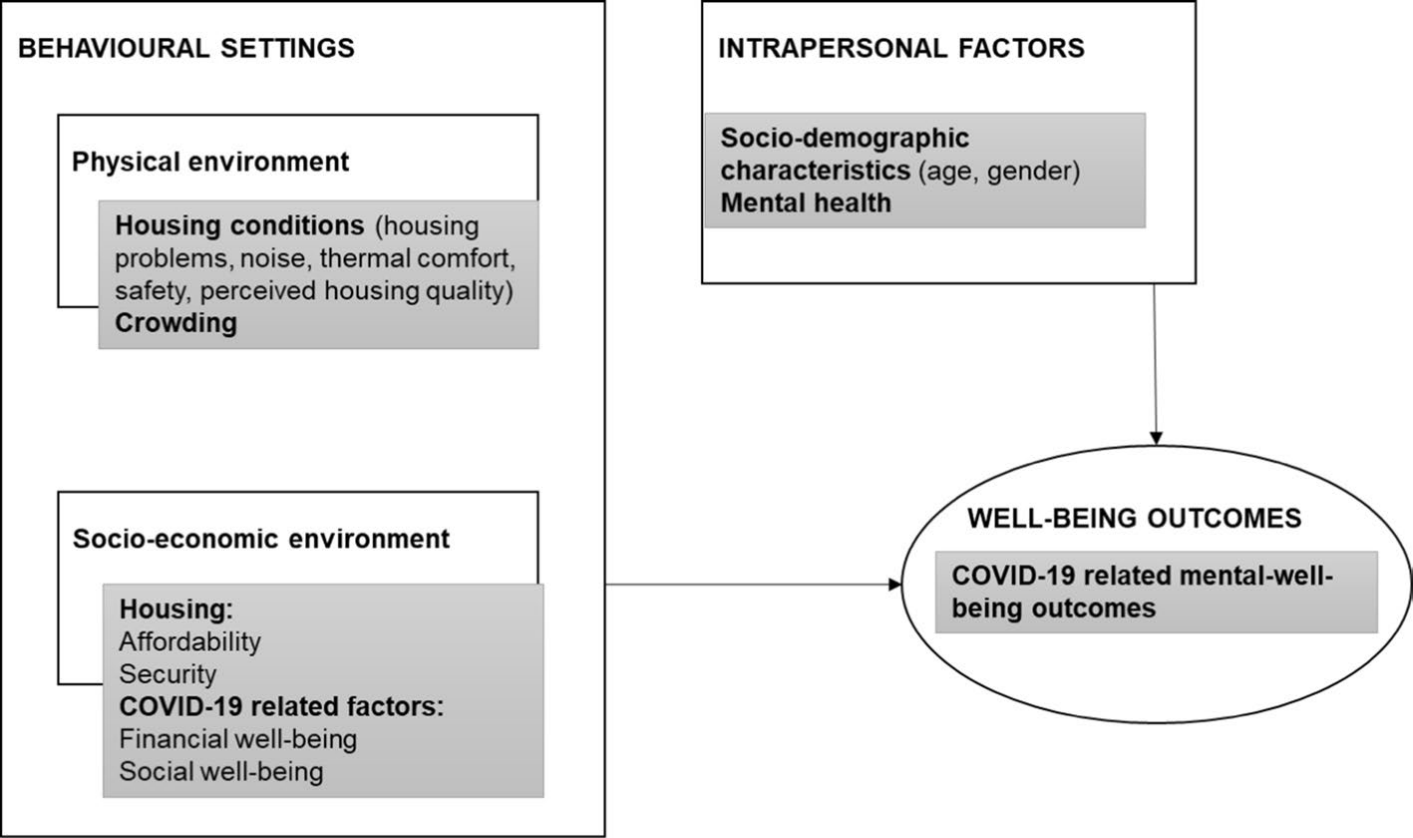
Social ecological model in this study. Grey boxes show how the model is considered in this study

We hypothesise that physical housing conditions (such as noise or thermal comfort), affordability and security, as well as common policy response to the COVID-19 crisis, contributed to COVID-19-related mental well-being. However, it is important to link these with social and economic circumstances. Thus, we rely on social-ecological (SE) model for health promotion (Stokols, 1992). SE model (Stokols, 1992) connects built environment and social environment while predicting behavioural outcomes. SE model has gained prominence in promoting well-being because ‘peoples’ transactions with their physical and socio-cultural environments’ (Sallis et al., 1998: 380) together with intrapersonal variables have proven to influence their mental, social and physical health. The underlying assumption of SE model is that behavioural settings or environments restrict or promote certain behaviours and thus health outcomes (Sallis et al., 1998). This implies that environmental and policy variables ‘can add explanatory value above that provided by intrapersonal and interpersonal factors’ (Ibid: 380). COVID-19 changed peoples’ normal behaviours, but also influenced their behavioural settings, such as the use of physical environments (e.g. working from home, lockdowns) and social environments (e.g. meeting with peers). Housing is central in our study as stay at home orders were main policy intervention to deal with the crisis. SE-model interrelates behaviour settings that are physical environments (e.g. architecture, geography and technology), social environments (e.g. culture, economics and policy) and personal attributes (e.g. psychological dispositions and behavioural patterns) when predicting health and well-being behaviours. Figure 2 shows that in our study, housing conditions and crowding (measured with number of people in home) are considered as physical environment, housing affordability and tenure security are housing related socio-economic factors, and COVID-19 specific financial and mental hardships are another socio-economic contributor. Together with personal socio-demographic characteristics and perceived mental health, these contribute towards peoples’ well-being outcomes.

First, COVID-19-related worry/anxiety and loneliness/isolation (dependent variables) were compared by household types. Then, descriptive statistics of independent and dependent variables and covariates were run.

Finally, two models were developed, one for worry/anxiety and another for loneliness/isolation. Binary logistic regression analysis was used to predict the dependent variables. Final models present significant variables only; non-significant predictors were excluded from the modelling throughout the analyses, to ensure model parsimony and enable ready replicability of the study methodology. All statistical procedures were carried out using IBM SPSS Statistics 27 software.

## Results

### Descriptive statistics of the sample

The ARHCD participants who resided in shared living arrangements reported higher levels of worry and anxiety due to COVID-19 (58.8%) compared to participants in other household structures. See Table 1. These findings were similar for loneliness and isolation.

**Table 1:** COVID-19-related change in mental well-being by household type

Mental well-being outcome	Couple with no children (*n* = 2984)	Couple with children (*n* = 3538)	One parent family (*n* = 1030)	Single person, living alone (*n* = 2159)	Shared living arrangement (*n* = 1908)
*Worry/anxiety*					
Yes	1368 (50.3%)	1509 (45.6%)	465 (50.3%)	882 (46.5%)	988 (58.8%)
No	1353 (49.7%)	1800 (54.4%)	460 (49.7%)	1013 (53.5%)	693 (41.2%)
*Loneliness/isolation*					
Yes	934 (34.3%)	973 (29.4%)	377 (40.8%)	834 (44.0%)	801 (47.7%)
No	1787 (65.7%)	2336 (70.6%)	548 (59.2%)	1061 (56.0%)	880 (52.3%)

Difference between the overall number of participants in each household type and the sum of number of respondents in worry/anxiety and loneliness/isolation groups reflect missing responses

The majority of the participants in shared living arrangement were female (61.9%), aged 18–29 years (54.1%) and 41.4% perceived that their mental health was average or poor, see Table 2.

**Table 2:** Socio-demographics of participants living in shared arrangement

Socio-demographic characteristics	Participants in shared living arrangement
*Gender*	
Male	721 (38.1%)
Female	1172 (61.9%)
*Age*	
18–29	1028 (54.1%)
30–49	648 (34.1%)
50+^a^	224 (11.8%)
*Perceived mental health*	
Excellent or very good	496 (26.2%)
Good	616 (32.5%)
Average or poor	784 (41.4%)

^a^Of these, 182 (9.6%) were aged 50–64 years and 42 (2.2%) were aged 65 +

Overall, little more than half of the sample (57.0%) found that their home was in excellent or good condition, but 43.1% perceived that their home had a negative effect on their mental health, see Table 3. 57.0% of participants were asked to work from home (WFH) due to COVID-19 crisis and 54.0% of participants found that their home was fairly adequate and 32.0% that it was not adequate for working from home. The majority (68.7%) lived in a house and 62.6% found that their rental housing was affordable or very affordable.

**Table 3:** Descriptive statistics of independent variables

Independent variables	Participants in shared living arrangement
*Housing conditions*	
Count of housing problems	
0	606 (31.8%)
1	371 (19.4%)
2	320 (16.8%)
3 or more	611 (32.0%)
Warm during winter	
Yes^a^	1412 (75.0%)
Count of security measures	
0	448 (23.5%)
1	554 (29.0%)
2	542 (28.4%)
3 or 4	364 (19.1%)
Overall condition of home	
Excellent or good	1085 (57.0%)
Average	644 (33.8%)
Poor or very poor	176 (9.2%)
Housing effect to mental health	
Positive effect	357 (19.1%)
Negative effect	806 (43.1%)
No effect	709 (37.9%)
Adequacy of home for WFH^b^	
Very adequate	133 (14.0%)
Fairly adequate	511 (54.0%)
Not adequate	303 (32.0%)
*Housing attributes*	
Dwelling type	
House	1,299 (68.7%)
Apartment	591 (31.3%)
No of bedrooms	
0–2	608 (32.1%)
3	770 (40.7%)
4+	516 (27.2%)
Years in building	
Less than 1 years	744 (39.1%)
1 to less than 2 years	516 (27.1%)
2+ years	644 (33.8%)
Affordability	
Very affordable or affordable	1131 (62.6%)
Neither affordable nor unaffordable	499 (27.6%)
Unaffordable or very unaffordable	177 (9.8%)
No of adults in home	
2	719 (39.0%)
3	648 (35.2%)
4 or more	476 (25.8%)
No of children in home	
0	1669 (89.5%)
1	93 (5.0%)
2 or more	102 (5.5%)
*COVID-19-related measures*	
Withdrawn super funds	
Yes^a^	280 (17.1%)
Used savings	
Yes^a^	712 (43.2%)
Rent had become unaffordable	
No^a^	1522 (90.5%)
Find housemate	
No^a^	1491 (88.7%)
Seek government assistance	
No^a^	1335 (79.4%)
Seek additional work	
No^a^	1458 (86.7%)
Requested to WFH^b^	
Yes^a^	954 (57.6%)
Difficulties in personal relationships	
No^a^	1199 (71.3%)
Loss of self-esteem	
No^a^	1128 (67.1%)
Experienced helplessness	
No^a^	1152 (68.5%)
Mental health change	
Decreased	1082 (66.4%)
No change	398 (24.4%)
Increased	150 (9.2%)

^a^Binary variable (yes/no scale)

^b^WFH refers to work from home

### Worry and anxiety due to COVID-19

The first model predicted worry and anxiety as a result of COVID-19, see Table 4. Binary logistic regression model was significant, Nagelkerke *R*^2^ = 0.451.

The only housing conditions variable that was significant was count of housing problems. However, only overall contribution of the multi-level variable was significant (*p* < 0.05), and the exact number of housing problems did not contribute to the model. None of the housing attributes were significant for predicting the model.

Participants who were requested to WFH due to COVID-19 were 1.35 times more likely to feel worried and anxious compared to participants who were not asked to WFH. Participants who reported having no difficulties in personal relationships, had not experienced increased loneliness/isolation or had not experienced helplessness due to COVID-19 were less likely to experience increased worry and anxiety (0.50, 0.47 and 0.27 times, respectively). Spearman correlation coefficients between worry/anxiety and these variables were weak, *r* ≤ 0.40. Respondents who reported having no change in mental health were 0.36 times less likely to experience worry and anxiety than those who perceived that their mental health had increased. Spearman correlation coefficient was 0.41.

In terms of financial well-being, participants who did not seek government assistance had less chance (0.67 times) of experiencing worry and anxiety compared to participants who had to seek assistance. Age group was not a significant model predictor. Males were less likely (Exp (B) = 0.63) to experience worry and anxiety compared to females. Finally, respondents who reported having excellent/very good or good mental health were less likely to experience worry and anxiety (0.39 and 0.63 times, respectively) compared to participants who reported poor mental health. Spearman’s correlation coefficient between the two was 0.36 (Table 4).

**Table 4:** Model 1, Worry and anxiety

Independent variables	Exp (*B*)	SE
Count of housing problems	**	
0	.748*	.164
1	1.111	.187
2	.705*	.190
3+ (ref)		
Requested to WFH due to COVID-19 (Yes)	1.346**	.139
Difficulties in personal relationships (No)	.501***	.167
Experienced loneliness/isolation (No)	.472***	.138
Experienced helplessness (No)	.272 ***	.173
COVID-19-related change in mental health	***	
Decreased	1.456*	.212
No change	.363***	.232
Increased (ref)		
Seek government assistance (No)	.668**	.174
Age group		
18–29	1.026	.223
30–49	1.075	.228
50+ (ref)		
Gender (Male)	.631***	.132
Perceived mental health	***	
Excellent or very good	.392***	.179
Good	.631**	.151
Average or poor (ref)		
Constant	23.367***	.375

***p < 0.01, **p < 0.05, *p < 0.1

### Loneliness and isolation due to COVID-19

The second model predicted higher levels of loneliness and isolation due to COVID-19, see Table 5. The model was significant, Nagelkerke *R*^2^ was 0.392. None of the housing conditions variables were significant for predicting COVID-19-related loneliness and isolation. Having two people living together (including reference person) increased chances of experiencing loneliness and isolation by 1.38 times compared to participants who lived with four or more people. Participants who were asked to WFH were 1.37 times lonelier and isolated compared to those who were not asked to WFH. Respondents who reported no increased worry and anxiety, no difficulties in personal relationships, no loss of self-esteem and who hadn’t experienced helplessness were less likely to experience loneliness and isolation (0.50, 0.46, 0.43 and 0.52 times, respectively). Spearman correlation coefficients between loneliness/isolation and these variables were weak, *r* ≤ 0.38. Participants who found that their mental health had decreased due to COVID-19 had 2.35 chances to feel lonelier and more isolated than those whose mental health had increased. Spearman’s correlation coefficient between loneliness/isolation and mental health change was 0.36. Respondents who did not have to find housemates were 1.52 times more likely to experience loneliness and isolation compared to those who had to find housemates due to financial reasons.

In terms of covariates, age group was not a significant model predictor, however, males had less chance (0.67 times) of experiencing loneliness and isolation compared to females. Finally, participants who reported excellent or very good mental health had lower odds (0.42) of experiencing loneliness and isolation compared to those who considered their mental health as average or poor. Spearman’s correlation coefficient between the two was 0.31 (Table 5).

**Table 5:** Model 2, Loneliness and isolation of participants in shared living arrangement

Independent variables	Exp (*B*)	SE
No. of adults	**	
2	1.376**	.161
3	.932	.159
4 or more		
Requested to WFH due to COVID-19 (yes)	1.366**	.134
Experienced worry/anxiety (no)	.497***	.141
Difficulties in personal relationships (no)	.469***	.147
Experienced loss of self-esteem (no)	.427***	.144
Experienced helplessness (no)	.523***	.147
COVID-19-related change in mental health	***	
Decreased	2.351***	.235
No change	1.226	.264
Increased (ref)		
Had to find housemate (no)	1.523**	.197
Age group		
18–29	1.225	.220
30–49	.947	.223
50+ (ref)		
Gender (male)	.666***	.130
Perceived mental health	***	
Excellent or very good	.416***	.185
Good	.959	.143
Average or poor (ref)		
Constant	2.045*	.371

***p < 0.01, **p < 0.05, *p < 0.1

## Discussion and limitations

We hypothesised that housing conditions (built environment) and housing affordability, security and COVID-19-related financial and mental well-being (socio-economic environment) combined with personal characteristics, contribute towards COVID-19-related mental well-being outcomes, based on socio-economic theory. This hypothesis was only partially supported, as we did not find strong evidence that physical environment (housing conditions) contributes to crisis-related mental well-being. Socio-economic environment and especially COVID-19-related mental well-being variables, however, were major contributors. Other studies have found that the pandemic had a negative impact on peoples’ mental health. Butterworth et al. (2022), for example, estimated that COVID-19-related restrictions led to an additional 2.6% of people being identified with a mental disorder in Victoria (Australia). This indicates that direct policy response (part of social environment) may play a critical role in crisis support. During the COVID-19 pandemic, the Australian Government set up mental health support phone lines and increased the number of subsidised psychological therapy sessions available to each person (Commonwealth of Australia Department of Health, 2022). Indeed, the number of mental services processed nationally increased since the beginning of the pandemic, peaking in August 2021 with 319,648 services processed within one week, compared to 246,564 services processed in similar time period in 2019. This was the time period with severe lockdowns in three Australian states and territories (Australian Institute of Health & Welfare, 2022). Compared to March 2019, the proportion of mental health related prescriptions in Australia increased by 18.6% in March 2020 when the first nationwide lockdown was announced (Australian Institute of Health & Welfare, 2022). Our study found that COVID-19-related financial well-being was an important mental well-being contributor. Participants who did not have to seek financial assistance from the government due to COVID-19 were less likely to feel worried and anxious. This is aligned with previous studies that have concluded that financial hardship affects mental health negatively (Kiely et al., 2015). Additionally, Botha et al. (2020) found that participants who experienced financial stress during the pandemic in Australia had four times higher mental distress levels compared to those who did not experience financial distress. In terms of intrapersonal variables, our study found that males were less likely to have negative mental health outcomes compared to females. This is aligned with numerous studies carried out in Australia, finding that women experienced higher levels of mental distress compared to men (i.e., Australian Bureau of Statistics, 2021; Biddle & Gray, 2021; Butterworth et al., 2022). Australian Bureau of Statistics (2021) and Butterworth et al. (2022) also found that younger age groups (18–34 years and 20–54 years, respectively) were more likely to feel distressed. Our study did not find that age was a significant model contributor, however, it is important to note that the majority of participants were young to middle aged adults, 54.1% of participants were aged 18–29 years and 88.2% were aged18-49 years. It would be interesting to study the causal effect of behavioural environment (physical and socio-economic) on people’s mental well-being and how it differs after the pandemic. A longitudinal study could investigate this further.

Our study found that shared living renters who were asked to work from home felt more lonely and isolated (1.4 times) and were more worried and anxious (1.4 times) compared to participants who did not have to work from home. Blanc and Scanlon Bradley (2022) studied working from home in shared houses in London during COVID-19 and found that most houses were not built and suitable for that purpose, mainly due to inappropriate design, lack of space and number of people living together. The same study found that participants lacked privacy and faced difficulties focusing on work when multiple people had to use same space for working (Ibid.). This might be one reason why the participants in our study had worse mental well-being outcomes. Additionally, pre-pandemic studies took it as a norm that the shared housing residents did not work from home (Blanc & Scanlon Bradley, 2022; Heath et al., 2017), this might mean that the participants in our study missed their previous lifestyle and social connections. Moreover, considering the importance of COVID-19-related mental and financial well-being measures, those participants may have been worried about their employment security. A qualitative study could explore this further.

The COVID-19 pandemic has influenced the role and meaning of home due to the obligation to spend more time in it; consequently, interpersonal relationships have also had a greater relevance (Rogers & Power, 2020). In a context of limited or delayed government support and reduced income due to the pandemic, people's mental well-being is more than ever linked to individuals who can be personally interacted with (Horne et al., 2020; Oswald et al., 2022). A surprising finding of our study was that people who lived with one other person had increased chance of experiencing loneliness and isolation compared to those in a household of four or more people. Potentially, living with more people provides a better social environment due to the variety of interactions possible, whereas living with only one person in a lockdown situation could strain even the best of friendships and create loneliness. Indeed, evidence has been found that shared living arrangements can generate a series of positive outcomes or coping mechanisms as catalysts for social capital, which is built by actions of mutual support and co-living (Leviten-Reid & Matthew, 2018). Social capital is based on relationships of trust and can be generated in situations that require offering or providing support (Markle et al., 2015). During the lockdowns, social interaction and support provided by people who shared housing reduced their perception of loneliness and isolation (compared to people who live alone), as reflected in the results of this study. Our results are consistent with other forms of shared housing, like cohousing communities (Izuhara et al., 2022), housing cooperatives (Guity-Zapata et al., 2023), multifamily housing (Ghimire et al., 2021), that indicate that living with more people during the pandemic contributed to the reduction of mental health issues. The results of this study indicate that the number of adults at home during pandemic-like crisis events can be both positive for relationship building and negative due to space constraints and disruptions. Understanding these effects via qualitative analyses would be a productive line of future inquiry.

It would be interesting to know if the isolation/loneliness and anxiety/worry experienced by participants influenced their future housing choices. In Blanc and Scanlon Bradley (2022, p.14) study of adults in shared housing in London, participants reported that lockdown experiences had changed their housing aspirations; for example, 18% became more motivated to leave shared accommodation and 16% placed a higher priority on having outdoor space at their next dwelling. While we did not ask our participants about their aspirations, considering the results of our survey, they may want to live with more or fewer people than previously, or they may critically evaluate future housing for its work from home capabilities. However, the ability to choose housing that matches one’s new, pandemic-era aspirations is entirely dependent on income, whether one is tied to a particular area through work or family, and the availability of housing stock. The shortage of affordable and appropriate housing for private renters, already a major issue raised by housing policy researchers and tenant advocates (Gurran et al., 2020; Hulse et al., 2019), is affected in new ways by the pandemic (see e.g. Baker et al., 2020b). Policy responses must therefore take into account the crisis context, but also plan for beyond it. If a great number of Australians are trying to work in housing that does not easily accommodate working from home—whether through poor insulation, unreliable internet, overcrowding or other housing problems—then there could be implications for broader workforce productivity as well workers’ mental well-being. Housing specific policy implications of these findings include a need for crisis interventions to include a focus on housing affordability to enable households to meet housing costs without introducing additional distress to household members. They additionally include a need to focus on the amenity of housing and home to accommodate digital and other forms of connectivity associated with working from home and/or schooling and training in the home environment. Finally, the results of this study suggest that future supply of new housing or retrofit of existing stock be designed with a broad understanding of universal design principles such that sufficient space and amenity, as well as flexibility, is provided for multi-functional housing and living.

This study, as any study, has limitations. Firstly, ARHCD participants who reported living in shared housing arrangements were included in this study. We note that it is unknown whether participants in this household structure were related (e.g. siblings sharing home) or unrelated (e.g. strangers sharing home), as discussed earlier. Secondly, income is often considered as a covariate in well-being studies, however, ARHCD measured household income and it is unknown whether all participants in shared living arrangements could report income of all household members adequately. Additionally, some people in shared living might have individual budgets. This is a limitation of the study. Noting that perceived housing affordability was entered as a housing attribute in the modelling and this measure is additionally a proxy of individual financial circumstances. We also note that various studies use different housing conditions (and housing affordability, security and crowding) variables, e.g. floor size to measure dwelling size. The ARHCD data that is the most comprehensive national dataset of housing conditions to date however is necessarily also limited. ARHCD was a cross-sectional study meaning that data was collected at a single point in time from a large sample (Bryman, 2016: 53). While this research design is widely used and sufficient for establishing relationships between variables, we note its main limitation of ‘ambiguity about the direction of causal influence’ (Bryman, 2016: 53). We also note that the results may have been influenced by other pandemic effects that were not measured with the survey and considered in this paper. Finally, worry and anxiety (and loneliness and isolation) are often considered as separate constructs in well-being studies, this was further discussed in Sect. 3.

## Conclusion

The study concluded that count of housing problems was the only housing variable that contributed to COVID-19-related mental well-being (in the worry and anxiety model). Based on the socio-economic model, we expected that housing conditions, affordability, security and other housing attributes considered in this study would have more effect on mental well-being, as many Australians were forced to spend significant time at home. However, most (57.0%) participants perceived their home in excellent or good condition and 62.6% found that their rent is very affordable or affordable. The implication is that other shocks, such as COVID-19-related direct effects, were more likely to cause mental well-being issues. Number of people living together was the only housing attribute that contributed to the COVID-19-related mental well-being (loneliness and isolation model), indicating that household size has a potential to act as a protective mechanism against loneliness and isolation.

Participants who were requested to work from home had higher chance of experiencing lower mental well-being in both outcome variables compared to those who did not have to work from home. This means that shared housing might not be the best environment to support mental health when working from home. Early indications suggest that WFH will continue at least partially (Ziffer, 2022), meaning that the problems experienced by people WFH in shared living arrangements are likely to persist.

Our key finding—that direct COVID-19-related effects were more likely to cause mental health problems than other housing variables—should not be taken as a sign that problems for renters in shared living ended when the lockdowns did. As stated, WFH continues in some form, and other impacts of the pandemic are unlikely to disappear. The financial impacts of losing work for prolonged periods, and the mental health impacts of living through a pandemic, will linger for many people. People in shared living arrangements, who are often on low incomes, in precarious work or have insecure tenancies (Nasreen & Ruming, 2019, 2021; Parkinson et al., 2021; Raynor & Panza, 2021) may have less resources to aid their recovery. The pandemic is still not over, and therefore there is a need for policies to adapt to pandemic life and acknowledge that problems will be ongoing.

By examining the housing conditions and mental well-being of a small sector in Australia—people in shared living arrangements in private rental housing—we have illuminated how pandemic-era policy measures, such as lockdowns, can negatively impact people’s mental well-being. However, policy measures also have the potential to help people recover from these impacts, if the policies acknowledge that the effects of the pandemic are complex, varied, and long-lasting. While our study focused on Australia, we suggest our results have relevance for the many countries that implemented lockdown restrictions during the pandemic and are experiencing a growth in shared housing arrangements.

## Data Availability

The datasets analysed during the current study are available in the Australian Data Archive Dataverse system repository: https://dataverse.ada.edu.au/dataset.xhtml?persistentId=doi:10.26193/IBL7PZ.
